# The Preparatory Activation of Guidance Templates for Visual Search and of Target Templates in Non-Search Tasks

**DOI:** 10.5334/joc.341

**Published:** 2024-01-10

**Authors:** Gordon Dodwell, Rebecca Nako, Martin Eimer

**Affiliations:** 1 Department of Psychological Sciences, Birkbeck, University of London, London, UK

**Keywords:** Attentional templates, attentional control, N2pc component, visual search

## Abstract

Representations of task-relevant object attributes (attentional templates) control the adaptive selectivity of visual processing. Previous studies have demonstrated that templates involved in the guidance of attention during visual search are activated in a preparatory fashion prior to the arrival of visual search displays. The current study investigated whether such proactive mechanisms are also triggered in non-search tasks, where attentional templates do not mediate the guidance of attention towards targets amongst distractors but are still necessary for subsequent target recognition processes. Participants either searched for colour-defined targets among multiple distractors or performed two other non-search tasks where imperative stimuli appeared without competing distractors (a colour-based Go/NoGo task, and a shape discrimination task where target colour was constant and could thus be ignored). Preparatory activation of colour-selective templates was tracked by measuring N2pc components (markers of attention allocation) to task-irrelevant colour singleton probes flashed every 200 ms during the interval between target displays. As expected, N2pcs were triggered by target-coloured probes in the search task, indicating that a corresponding guidance template was triggered proactively. Critically, clear probe N2pcs were also observed in the Go/NoGo task, and even in the shape discrimination task in an attenuated fashion. These findings demonstrate that the preparatory activation of feature-selective attentional task settings is not uniquely associated with the guidance of visual search but is also present in other types of visual selection tasks where guidance is not required.

## 1 Introduction

Mechanisms of selective attention prioritize the processing of task-relevant objects, to facilitate their detection and recognition, and to ensure the speedy selection and execution of adaptive responses to these objects. To achieve these goals, attentional selectivity must be controlled by internally represented information about target-defining attributes (“attentional templates”; see Duncan & Humphreys, 1989). Over the past three decades, much research on attentional control has investigated the nature of these templates, their neural basis, their association with visual working memory, and their interaction with incoming sensory input to facilitate the selection of relevant information (see Desimone & Duncan, 1995; Olivers et al., 2011; Eimer, 2014, 2015, for reviews).

It has been well documented that attentional templates can guide the allocation of attention in tasks where relevant target objects appear together with multiple irrelevant distractors in the same display. In such visual search tasks, the features of target objects are usually known in advance, and this knowledge can be used to bias attentional processing towards objects with template-matching features (see Duncan, 2006; Wolfe, 2007, 2021; Eimer, 2014; Ort & Olivers, 2020, for details). Importantly, such search templates can be activated in a preparatory fashion prior to the presentation of a search display (e,g., Chelazzi et al., 1998; Grubert & Eimer, 2018, 2020). This preparatory activation of search templates is clearly adaptive in search tasks where a target appears together with multiple competing distractors, particularly when limited time is available to find this target (e.g., with short search display durations). Under these conditions, the fact that a template is already active when the search display appears maximizes the chances that attention is successfully allocated to a target object, so that this object can then be selected and identified.

Importantly, the guidance of attention towards possible target objects in multiple-item search displays is not the only function that is associated with attentional templates. Once attention has been allocated to an object, and after this object has been selected and encoded into working memory, it still needs to be recognized. During object recognition, incoming sensory representations are compared to stored memory representations of the target object and its response-relevant features. As a result, this object is recognized as a target or nontarget, and the attributes that determine response selection are identified. While these memory representations are also commonly referred to as “attentional templates”, it is important to distinguish templates that mediate attentional guidance and templates that mediate object recognition. These two types of templates have different functions and operate at different stages of visual processing.

There are situations, such as when observers simply report the presence or absence of a specific predefined target object in a search display, where guidance and recognition can be based on a single target template. However, there are other tasks where two distinct templates need to be employed. One such case is visual search for compound targets (Duncan, 1985; Olivers & Meeter, 2006) where target-defining and response-relevant attributes are distinct. For example, in a task where observers must find a red target object and then report whether it is a letter or digit, guidance is based on colour, and recognition on alphanumeric category. Another case is hybrid visual/memory search (Wolfe, 2012), where observers memorize a large set of possible target objects, then report the presence of a specific member of this set in each search display (e.g., one exemplar of a set of *n* different animals). Hybrid search can be performed with memory sets of 100 objects or more, far exceeding the capacity of working memory, wherein search templates responsible for attentional guidance are assumed to be represented (Olivers et al., 2011). Based on such observations from hybrid search, the most recent version of the influential Guided Search model (Guided Search 6.0; Wolfe, 2021) postulates the existence of two distinct search templates – a guiding template that biases the initial allocation of attention towards template-matching features, and a target template that mediates subsequent target recognition processes. Finally, there are also many attentional tasks where target objects are presented without competing distractors, so that no guidance is needed. In such non-search tasks, target templates are still required to identify a target object and select its assigned response, but guiding templates are obviously not involved.

While there are good reasons to assume that the attentional templates which control the guidance of visual search and the templates that enable the recognition of selected objects are distinct, differences in the way these two types of templates operate have so far not been investigated systematically. For example, it is possible that guidance and target templates fundamentally differ with respect to their respective temporal activation profiles. Guiding templates are known to be activated in a preparatory fashion (e.g., Chelazzi et al., 1998; Grubert & Eimer, 2018, 2020; see also Olivers et al., 2011). This enables these templates to efficiently bias the allocation of attention during the early parallel processing of visual input towards candidate target objects. In contrast, the templates that are involved in target recognition only become relevant once a particular object has been selected and encoded. Given these contrasting temporal constraints, preparatory activation may be uniquely associated with guiding templates and standard visual search tasks where target objects must be located among multiple competing nontargets. Correspondingly, no such preparatory processes may be elicited in non-search tasks, where task-relevant objects are not accompanied by distractors and thus no guidance of attention is required. In such tasks, the activation of target templates may not occur proactively during task preparation, but in a purely reactive fashion once sensory input has been received.

In a series of recent studies in our lab (Grubert & Eimer, 2018, 2020), we used electrophysiology to measure the preparatory activation of guidance templates. In these experiments, event-related potential (ERP) markers of these activation processes were obtained during the period when observers prepared for an upcoming search display that included a colour-defined target object among multiple differently coloured distractors. To track the activation state of target-colour guidance templates, a rapid serial probe presentation (RSPP) procedure was used where series of irrelevant probe displays appeared at regular intervals (every 200 ms) during the interval between two successive search displays. Probes contained a colour singleton item that could match the colour of the target. The critical assumption was target-matching colour singleton probes will capture attention only when they appear during the period when a corresponding colour template is activated. The absence of attentional capture by these probes thus indicates that this template is inactive at the time when the probe is presented. To track probe-induced attentional capture, EEG was recorded during task performance, and N2pc components (an ERP marker of the allocation of attention to candidate target objects; e.g., Eimer, 1996; Luck & Hillyard, 1994; Woodman & Luck, 1999) were measured in response to each successive probe display. Previous work has demonstrated that successive attentional allocation processes as indexed by the N2pc are triggered in parallel and independently when displays are presented in rapid succession (Eimer & Grubert, 2014; Grubert & Eimer, 2015; Grubert et al., 2017; Jenkins et al., 2016). Thus, even when probes appear in rapid succession, N2pc components indicative of search template activation can still be reliably obtained for each individual probe. Singleton probes that matched the target colour triggered N2pc components from about 1000 ms prior to the onset of the next search display, but not earlier, indicating that target templates were activated in a transient fashion during each search preparation period. Probes that did not match this template did not trigger reliable N2pc components. We also demonstrated that this temporal activation profile was sensitive to the expected arrival time of the next search display. Reliable probe N2pcs were already elicited soon after the offset of the preceding search display in blocks where the interval between two search displays was short (1000 ms), but much later during the preparation period when this interval was longer (2400 ms).

**Figure 1:**
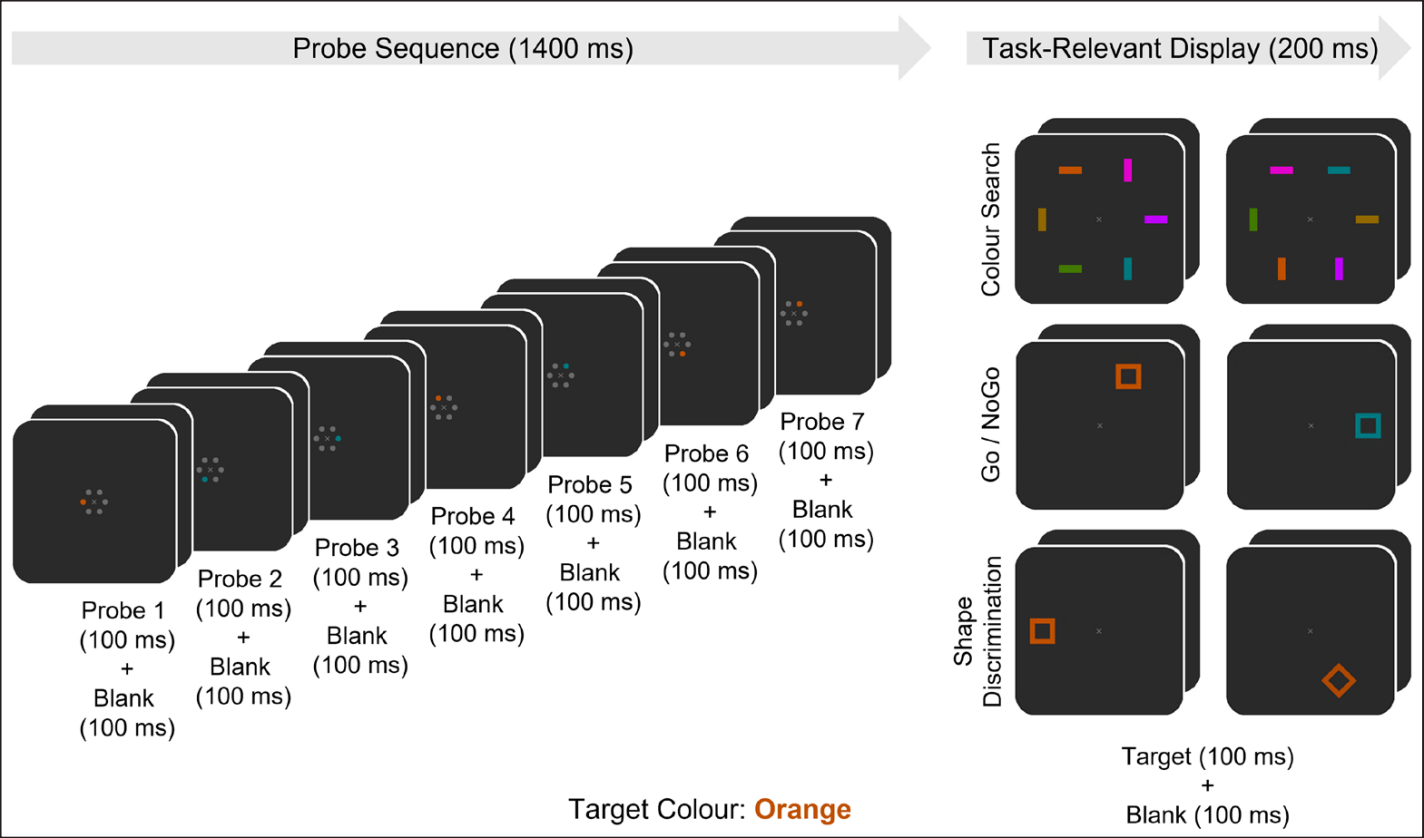
Time course of the per-trial stimulus-events occurring in each task (Colour Search, Go/NoGo, Shape Discrimination). A series of 12 consecutive trials occurred in each block of the experiment, with 24 blocks per task, and each block beginning with the pseudo-random assignment of a target colour (in this example, orange). Trials began with a sequence of seven probe-displays (100 ms), each followed by a blank screen (100 ms). Every probe display included either a target colour or non-target colour singleton probe, which was pseudo-randomly located amongst five grey placeholders. Following the probe sequence, a task-relevant display appeared (100 ms), followed by a blank screen (100 ms). In the Search task, the task-relevant display included one target colour bar amongst five other unique non-target colour bars, all of which were pseudo-randomly located and could be either horizontally or vertically oriented. Participants were instructed to indicate the orientation of the target colour bar. In the Go/NoGo task, the task-relevant display included a single unfilled square pseudo-randomly located at one of six potential locations. The square could appear in either the target colour or a different non-target colour. Participants were instructed to only respond to target colour items and to withhold responses when the item appeared in the other non-target colour. In the Shape Discrimination task, the task-relevant display included a single unfilled shape pseudo-randomly located at one of six potential locations, which always appeared in the target colour and could be oriented at either 0° (“a square”) or 90° (“a diamond”). Participants were instructed to indicate the shape of the item.

These results provide direct electrophysiological evidence for the transient activation of guidance templates during the preparation for an upcoming search episode. The goal of the current study was to find out whether such a proactive temporal profile is exclusively associated with tasks that require attentional guidance. If this was the case, no such preparatory activation should be observed under conditions where attention does not have to be guided to a target object because no competing distractors are present. In contrast, if not only guidance templates but also target templates can operate in a proactive fashion, evidence for the advance activation of these templates should be found in non-search tasks. To investigate these alternative possibilities, we tracked the temporal activation profiles of colour-selective attentional templates separately and independently for tasks where these templates were either involved in the guidance of attention or in the recognition of target objects. We employed RSPP procedures that were analogous to those used in previous work (Grubert & Eimer, 2018, 2020). Probe displays containing one of two possible colour singletons were presented every 200 ms during the interval between task-relevant displays (see Figure 1). N2pc components were again measured to each successive probe display, as markers of attentional capture indicating that a corresponding colour-specific template is active. Critically, participants performed three different tasks (see Figure 1), where colour was relevant either only for guidance, only for target recognition, or neither for guidance nor recognition.

In the Colour Search task, a target object (a horizontal or vertical bar) in a pre-defined colour appeared together with five other distractor bars in five different colours. Each task-relevant display was presented for 100 ms, and participants had to report the orientation of the target bar. Probe displays either contained a singleton that matched the target colour, or a singleton in a different non-target colour, with equal probability (see Figure 1). In this task, colour-based guidance was required to allocate attention rapidly to the target bar and not to any of the distractors. As response selection was based on orientation, no colour-selective target template was involved. Here, a colour-specific guidance template should be proactively activated during the preparation period, as reflected by a temporal pattern of N2pc components to target colour probes similar to the pattern observed in our earlier studies (Grubert & Eimer, 2018, 2020). In contrast, no reliable N2pcs should be elicited by non-target colour probes.

To assess whether attentional templates are only activated in a preparatory fashion when attentional guidance is required, participants also performed two other non-search tasks. In contrast to the Colour Search task, the task-relevant displays in these two tasks only contained a single object, without any accompanying distractors (see Figure 1). In the Go/NoGo task, participants had to respond when the object was presented in a pre-defined target colour, and refrain from responding when this object had a different colour. The colour singleton probes matched one of these two colours, with equal probability. Participants therefore had to employ a colour-specific template for recognition in order to discriminate Go and NoGo objects. However, and critically, in the absence of any competition by distractors in the task-relevant display, no colour-based guidance template would be required. If attentional templates are only activated in a preparatory fashion when these templates are needed to guide attention in visual search displays, no such proactive processes should be triggered in the Go/NoGo task, and this should be reflected by the absence of any probe-induced N2pc components. Alternatively, if attentional templates are still activated in a preparatory fashion even when they are only involved in target recognition but not in guidance, reliable probe N2pcs should be observed prior to the presentation of task-relevant displays in the Go/NoGo task, particularly for singleton probes that match the target colour. We also included a third task (Shape Discrimination), wherein the task-relevant displays again only contained a single object. This object always appeared in the pre-defined target colour, and participants had to report its shape (a square or a diamond). Singleton probes appeared either in the target colour or in a different colour. In this task, no attentional guidance was involved, and colour was also not required for target recognition, as response selection was based on shape. Therefore, no preparatory colour-specific guidance or target template activation processes should be triggered at all, and this should be reflected by the absence of N2pc components to any of the colour singleton probes.

## 2 Methods

### 2.1 Participants

The study was conducted with 19 participants, all of whom provided informed written consent prior to testing and were monetarily compensated. One participant’s dataset was excluded due to excessive ocular artefacts. The final sample therefore consisted of 18 participants (*M* = 28.7, *SD* = 7.3, ten female, three left-handed), all of whom reported normal or corrected-to-normal vision. The sample size of 18 was sufficient for appropriate counterbalancing of task order and exceeded the minimum of 15 estimated by an a-priori power analysis (*α* = 0.5, 1 – β = .90, *ƒ* = 0.85; G*Power; Faul, Erdfelder, Lang, & Buchner, 2007). The effect size used in this power analysis was based on the amplitude of N2pc components (main effect of probe laterality) triggered in response to colour singleton probe stimuli in a prior study employing a similar RSPP procedure (Grubert & Eimer, 2020). The study was conducted in accordance with the Declaration of Helsinki and approved by the Psychology Ethics Committee at Birkbeck, University of London.

### 2.2 Materials

Participants performed the experiment while seated in a dimly lit, sound-dampened, temperature-controlled, and electromagnetically shielded room. Stimuli were presented on a 24.5-in monitor (Zowie XL2546; TN panel, 1920 × 1080 resolution, 240 Hz refresh rate) at a viewing distance of approximately 100 cm. Presentation of the experiment was controlled by a dedicated PC running Windows 10 (Acer PO3-630; Intel Core i7-11700F, NVIDIA GeForce RTX 3070) and coded in Python (ver. 3.9.13), primarily using the Psychopy toolbox (ver. 2022.2.3; Peirce et. al., 2019).

The EEG data were continuously sampled at 1 kHz with active Ag/AgCl electrodes connected to a digital amplifier (ActiCAP slim/snap & BrainAmp DC; Brain Products GmbH, Gilching, Germany) placed over 61 scalp sites in accordance with the international 10–10 system. Three additional electrodes were placed on the inferior orbit of the left eye and the outer canthi of both eyes to monitor vertical and horizontal eye movements. Electrodes positioned over scalp sites AFz and FCz served as the online ground and reference, with electrode impedances kept at less than 10 kΩ. The raw EEG data was recorded using BrainVision Recorder (ver. 1.25.0001, Brain Products GmbH, Gilching, Germany) on a dedicated PC running Windows 10 (Dell Precision 5820, Intel Xeon W-2235, NVIDIA Quadro P2200) and digitally filtered during recording with a low-pass filter of 250 Hz and a high-pass filter of 0.01 Hz. The event timings were synchronized between the presentation and recording computers with a delay of no greater than 1 ms.

### 2.3 Stimuli and procedures

The experiment employed a rapid serial probe presentation (RSPP) paradigm where task-irrelevant probe displays including a lateralised colour singleton appeared every 200 ms during the interval between two successive task-relevant displays. There were three tasks (Colour Search, Go/NoGo, Shape Discrimination) which employed the same probe displays (see Figure 1, left side), but differed with respect to their task-relevant displays and instructions (Figure 1, right side). Each task included 24 blocks, with 12 trials per block, for a total of 288 trials per task. All stimuli were presented on a dark grey background (5.25 cd/m2; 0.285/0.347 CIE x/y).

Each block started with a 500 ms blank screen, followed by a grey fixation cross (0.2° × 0.2° visual angle; 20.5 ± 0.5 cd/m2; 0.293/0.340) which appeared at screen centre 500 ms prior to the series of trials, then remained on screen throughout the rest of the block. Each trial began with a sequence of seven consecutive task-irrelevant probe displays followed by a task-relevant display, where each display was presented for the first 100 ms of a 200 ms interval, resulting in a total trial duration of 1600 ms. Trials ran consecutively – that is, without pause between the end of one trial and the beginning of the next. Therefore, responses were given after each task-relevant display during the probe sequence of the following trial. A 13^th^ probe sequence was included after the 12^th^ trial in each block to maintain the perceptual consistency of the response windows, but data from these probes was not included in analysis.

Each probe display included a circular arrangement of six filled circles (0.3° visual angle ⌀) located 0.6° of visual angle from the fixation cross at screen centre and evenly spaced at 30°, 90°, 150°, 210°, 270°, and 330° relative to the positive y-intercept. One circle always served as the colour-singleton probe (e.g., “the probe”), appearing in one of two possible colours (the target colour or another non-target colour, see below). The remaining five circles were uniformly grey (20.5 ± 0.5 cd/m2; 0.293/0.340) and served as spatial placeholders to prevent any perceptual imbalances in the display. The colour and location of the probe was pseudo-randomized such that an equal number of target colour and non-target colour probes appeared at each of the six probe locations within all seven probe displays across each task. To avoid any effects of spatially specific sensory refractoriness on ERP responses to probe displays, two consecutive probes never appeared in the same location.

In each block, new target and non-target colours were selected from a set of six equiluminant colours (20.5 ± 0.5 cd/m2; orange: 0.513/0.436, gold: 0.432/0.497, green: 0.341/0.567, teal: 0.219/0.321, purple: 0.294/0.093, pink: 0.325/0.161). The assigned target colour was pseudo-randomized to ensure that each colour was selected for four of the 24 blocks within each task, to eliminate effects of colour preferences or differential ERP responses. To avoid systematic pairings between the target and non-target colours, the non-target colour was also pseudo-randomly selected per-block, excluding the target colour and that most proximal to it (e.g., avoiding gold with orange, green with teal, and purple with pink). The target colour for each block was indicated to the participant on an instruction screen presented prior to the start of each block (e.g., “TARGET COLOUR: BLUE”, with the word ‘blue’ appearing in the corresponding colour).

In the Colour Search task, the task-relevant display consisted of a circular arrangement of six filled rectangular bars (0.4° × 1.2° visual angle) located 3° of visual angle from the fixation cross at screen centre and evenly spaced at 30°, 90°, 150°, 210°, 270°, and 330° relative to the positive y-intercept. Each bar appeared uniquely in one of the six potential colours (including the target and non-target colours of the current block) and was either horizontally or vertically oriented. To eliminate orientation as a distinguishing factor, three randomly selected bars were oriented vertically and the other three were oriented horizontally. Participants were instructed to search the task-relevant display for the target colour bar, thus necessitating the activation of a colour-selective guidance template. They reported whether the target colour bar was vertically or horizontally oriented by pressing the up or down keys, respectively. The location and orientation of the target colour bar were pseudo-randomized such that it remained unpredictable in any given trial but appeared at each potential target location and in each orientation with equal probability across all blocks.

In the Go/NoGo task, the task-relevant display included a single unfilled square (1.3° × 1.3° visual angle outer area, 0.8° × 0.8° visual angle inner area), which appeared at one of six potential locations (3° visual angle from screen centre; 30°, 90°, 150°, 210°, 270°, and 330° to the positive y-intercept), in either the target colour or the non-target colour. Participants were required to respond (by pressing the up key) only when this item appeared in the target colour, and to refrain from responding when it appeared in the non-target colour. The location and colour of the task-relevant item was pseudo-randomized across all trials, so that across all blocks, an equal number of items in both the target and non-target colour would occur at each possible location.

In the Shape Discrimination task, the task-relevant display again included a single unfilled shape that appeared in the same dimensions and locations as in the Go/NoGo task. This shape was always presented in the target colour and could be oriented at either 0° (“a square”) or 90° (“a diamond”). Participants were instructed to indicate whether this item was a square or a diamond by pressing the up or down keys, respectively. The locations and shapes of these items were pseudo-randomized across all trials in the same way as the locations and colours of the analogous items in the Go/NoGo task.

### 2.4 EEG pre-processing

The EEG data were pre-processed using MNE Python (ver. 1.3.0; Gramfort et. al., 2013). The continuous data were first notch-filtered in 50 Hz intervals between 50 and 250 Hz to remove any potential line noise. The continuous data were then manually inspected to exclude any bad channels (*M* = 0.67, *SD* = 1.15), segments where substantial artefacts could be visually identified (*M* = 1.72%, *SD* = 2.08%), and those blocks where error rates exceeded 35% (*M* = 1.16%, *SD* = 2.83%; considered likely to be due to the participant responding to the incorrect target colour). Following manual inspection, an extended Picard independent component analysis (ICA) was performed across all channels of the continuous data (61 EEG + 3 EOG, excluding those previously identified as bad; 500 steps, convergence bound = 1 × 10^–7^). Components representing blinks and saccades were identified per subject based on an automatic correlational rejection provided by MNE and visual inspection of the individual time courses and topologies. Those components identified as EOG artefacts were then removed, prior to a back projection of the residuals. Following ICA, a 40 Hz low-pass FIR filter was applied before re-referencing all EEG signals to the 61-channel common average. The data were then segmented into 500 ms epochs relative to the onset of each probe display, including a 100 ms pre-stimulus baseline and a 400 ms post-stimulus time window. The epochs were then passed through an automatic amplitude-driven artefact rejection, based on a generalized ESD test with a significance threshold of 0.05 (Rosner, 1983), as implemented in the OHBA Software Library (OSL; https://ohba-analysis.github.io/). On average, this resulted in 1.4% (*M* = 32, *SD* = 22) of epochs being rejected per task across participants. From the remaining epochs, ERPs measured at lateral posterior electrodes PO7 and PO8 were then averaged for each of the three tasks, separately for each probe time (probe 1–7), probe colour (target colour or non-target colour), and probe laterality (appearing to the left or right of screen centre).

### 2.5 Behavioural analysis

For each participant, mean error rates (ERs) were first calculated block-wise to exclude those with an average ER exceeding 35% from further behavioural analysis (matching those excluded from ERP analysis mentioned prior). The ERs from the remaining blocks were then averaged per task. Mean response times (RTs) were then also calculated per task, excluding trials with erroneous responses, as well as those with RTs faster than 200 ms or slower than 1000 ms. The per-task ERs and RTs of each participant were then compiled and analysed within two separate one-way repeated measures ANOVAs to compare the behavioural effects of each task (Colour Search, Go/NoGo, and Shape Discrimination).

### 2.6 ERP analysis

ERP analyses were based on mean amplitudes calculated within an 80 ms time window beginning 180 ms after stimulus onset. This time window was derived from the 50% peak-amplitude onset and offset times of the grand-averaged N2pc observed at probe 7 across all three tasks. The N2pcs of probe 7 were selected for this purpose as they demonstrated the largest and most reliable negativities across all participants and conditions. This method of determining an analysis time-window is better tailored to the present data than generic standards (e.g., 200–300 ms post-stimulus) and is also analogous to previous studies that used similar probe presentation procedures (e.g., Grubert & Eimer, 2020).

N2pcs to target objects in the task-relevant display of the Colour Search task were evaluated with a t-test comparing contralateral and ipsilateral ERP mean amplitudes in the 180–260 ms post-stimulus interval. No target N2pcs were quantified in the other two tasks, as their task-relevant displays only ever contained a single item. Because this item was lateralized, it would not be possible to distinguish between target N2pc components and earlier ERP lateralisation associated with asymmetrical visual responses to the singular visual stimulus in the left versus right hemifield.

Probe N2pc components were evaluated by comparing ERPs at electrodes contralateral and ipsilateral to the side of the colour singleton in the probe display. Within each task, mean amplitudes of the contralateral and ipsilateral waveforms were first submitted to an overall 2 × 2 × 7 repeated-measures ANOVA with the factors Laterality (electrode contralateral vs ipsilateral to the side of the colour singleton probe), Probe Colour (target colour vs non-target colour singleton probe), and Probe Time (probes 1–7). A further 2 × 7 repeated-measures ANOVA was then performed separately for target colour and non-target colour probes, with factors Laterality and Probe Time. Follow-up pairwise Bayesian comparisons were also performed per probe colour between the contralateral and ipsilateral waveforms at each probe time, to substantiate the presence or absence any lateralisation. We opted to employ Bayesian analyses rather than classical frequentist statistics to assess individual probe N2pcs because they provide additional quantitative information about the strength of the evidence for the presence (or absence) of N2pcs at certain probe times. In all instances, this information was also corroborated by corresponding frequentist statistics (which are not reported).

Additionally, cluster-based permutation analyses (Maris and Oostenveld, 2007) were performed between the contralateral and ipsilateral waveforms of each successive probe, separately for the target and non-target coloured probes of all three tasks. Cluster-based permutation testing is a two-stage process that provides a non-parametric statistic which avoids the multiple-comparison problem when evaluating highly dimensional data and can indicate the probability of differences between EEG timeseries. In the *clustering* stage, a parametric test (e.g., a *t*-test) is performed between conditions at each timepoint and for each participant. From the resulting “map” of test statistics, adjacent values that exceed a pre-defined threshold (e.g., *t* = 1.96) are identified as “clusters”, which are then summed to provide a relative “cluster-statistic”. This is followed by an *inference* stage, wherein the identified clusters are evaluated against a distribution of cluster statistics found after randomly permutating the same timepoints from the participant-specific trial data a pre-defined number of times. For each cluster identified in the non-permuted data, a *p*-value is then calculated as the proportion of larger clusters found in the permutated distribution (see Sassenhagen & Draschkow, 2019). Here, we used 25,000 permutations and clustered timepoints where the contralateral waveform was significantly more negative than the ipsilateral waveform (one-tailed, alpha-level of 0.05).

## 3 Results

### 3.1 Behaviour

The ANOVA of RTs on trials with correct responses found a highly significant effect of Task (Colour Search, Go/NoGo, Shape Discrimination; *F* (2,34) = 187.00, *p* < 0.001, η2G = 0.72). RTs were slowest in the Colour Search task (*M* = 584 ms, *SD* = 62.13), intermediate in the Shape Discrimination task (*M* = 500 ms, *SD* = 81.47), and fastest in the Go/NoGo task (*M* = 344 ms, *SD* = 43.50). Pairwise comparisons between all three tasks confirmed that RTs were significantly slower in the Colour Search task as compared to both the Go/NoGo task (*t* (17) = 20.39, *p* < .001, *d* = 4.46) and the Shape Discrimination task (*t* (17) = 6.44, *p* < 0.001, *d* = 1.16). RTs were also slower in the Shape Discrimination task relative to the Go/NoGo task (*t* (17) = 12.13, *p* < 0.001, *d* = 2.37).

A corresponding pattern of results was found for ERs. There was a highly significant effect of Task (*F* (2,34) = 26.72, *p* < 0.001, η2G = 0.43), as incorrect responses were more frequent in the Colour Search task (*M* = 6.89%, *SD* = 2.86), intermediate in the Shape Discrimination task (*M* = 3.75%, *SD* = 3.34) and lowest in the Go/NoGo task (*M* = 1.29%, *SD* = 1.60). Pairwise comparisons between all three tasks indicated the ERs in the Colour Search task to be significantly higher than in both the Shape Discrimination task (*t* (17) = 3.89 *p* = 0.001. *d* = 1.01) and the Go/NoGo task (*t* (17) = 7.59, *p* < 0.001. *d* = 2.41). ERs observed in the Shape Discrimination task were also significantly higher than in the Go/NoGo task (*t* (17) = 3.24, *p* = 0.005. *d* = 0.94).

### 3.2 N2pc to target objects in the Colour Search task

**Figure 2:**
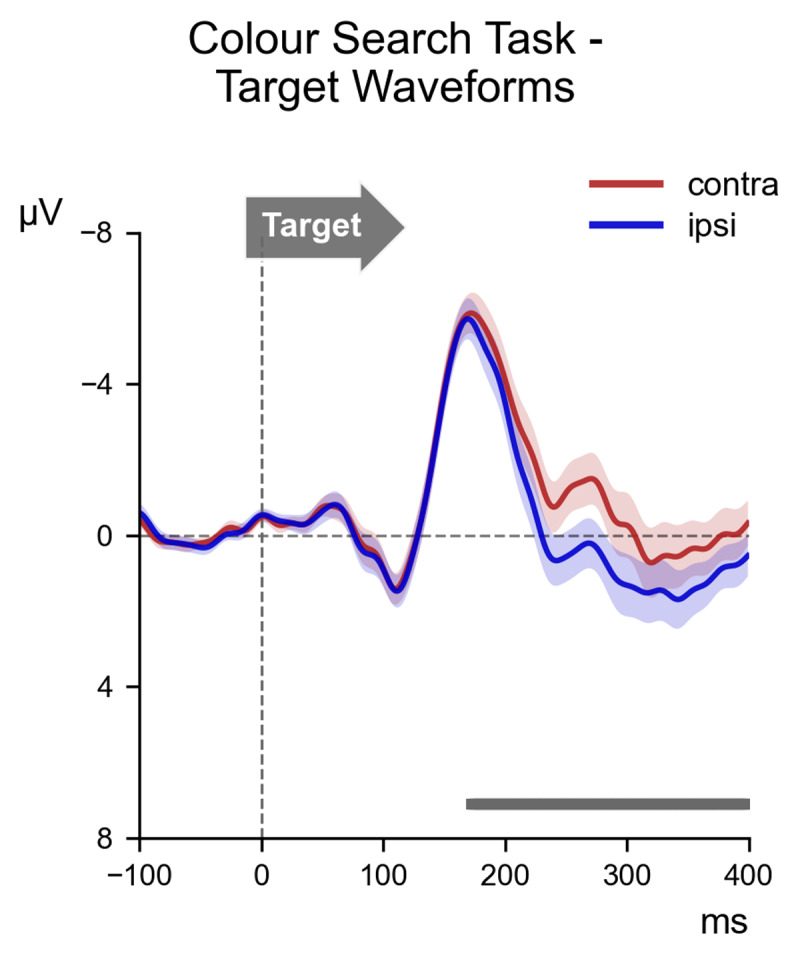
ERP waveforms elicited over scalp locations PO7/PO8, contralateral (red) and ipsilateral (blue) to the location of the target item in the task-relevant display of the Colour Search task. The waveforms are shown beginning 100 ms before the onset of the task-relevant display (serving as a baseline window) and proceeding for 400 ms thereafter. The contralateral waveform appears to become substantially more negative than the ipsilateral waveform. This observation was confirmed by permutation analysis, which indicated a large cluster (shown as a grey bar at the bottom of the frame) where the contralateral waveform was reliably more negative than the ipsilateral waveform, beginning approximately 170 ms after stimulus onset.

Figure 2 shows the contralateral and ipsilateral ERPs elicited in response to the task-relevant display in the Colour Search task. There was a significant amplitude difference between the contralateral (*M* = –2.67, *SD* = 2.90) and ipsilateral (*M* = –1.57, *SD* = 2.88) waveforms (*t* (17) = –7.14, *p* < 0.001, *d* = 0.38) in the 180–260 ms post-stimulus time window, confirming the presence of a reliable N2pc to search targets. This was corroborated by the permutation analysis, which identified a large cluster from approximately 170 ms post-stimulus onwards where contralateral ERPs were reliably more negative than ipsilateral ERPs (marked by the horizontal grey line in Figure 2).

### 3.3 Probe N2pcs

#### 3.3.1 Colour Search Task

**Figure 3:**
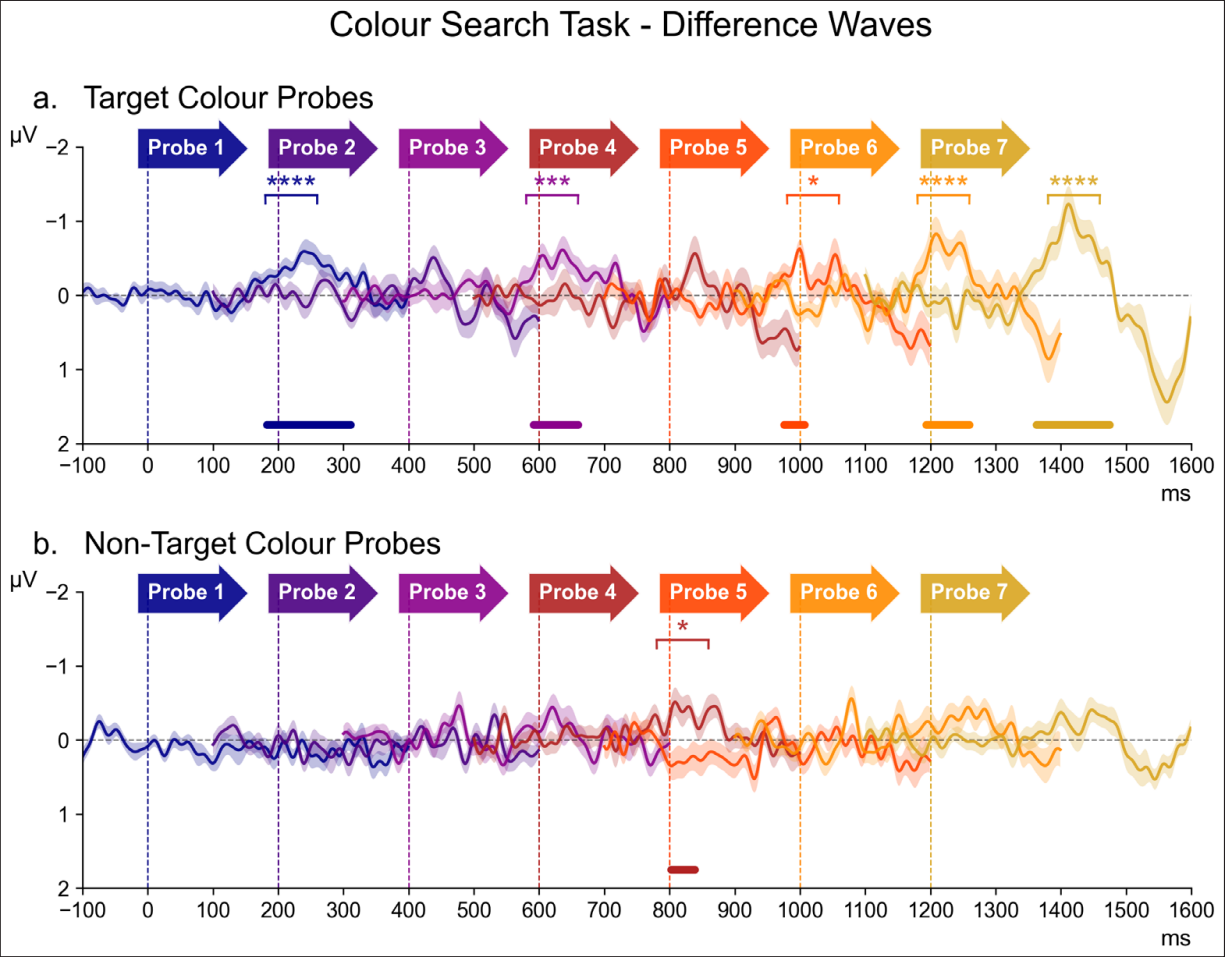
The series of difference waves (contralateral – ipsilateral waveforms) elicited in the Colour Search Task over scalp sites PO7/PO8, by target colour probes (panel a) and non-target colour probes (panel b). The onset latency of each probe is demarcated by a vertical dotted line in the colour of the probe label directly above. The waveform of each probe also appears in the corresponding colour, beginning 100 ms before the onset of the relative probe display (serving as a baseline window) and proceeding for 400 ms thereafter. Waveforms for which Bayesian analysis revealed contralateral negativity within the N2pc time window (180–260 ms after probe onset) are marked above with asterisks in the corresponding colour (* = *BF*_10_ > 3, ** = *BF*_10_ > 10, *** = *BF*_10_ > 30, **** = *BF*_10_ > 100). Similarly, portions of the waveforms wherein the permutation analysis detected significant clusters of contralateral negativity are marked below with horizontal bars in the corresponding colour.

Figure 3 shows contralateral minus ipsilateral difference waveforms for all seven successive probe displays in the Colour Search task, separately for the target colour and non-target colour probes (panels a and b). The contralateral and ipsilateral ERPs on which these difference waves were based are included in the appendix (Figure 6). In Figure 3, difference waves for each probe are colour-coded, and shown from 100 ms prior to 400 ms after probe onset, relative to a 100 ms pre-stimulus baseline, within the entire 1600 ms interval that separated two successive task-relevant displays. The onset of each probe is indicated by coloured arrows. Asterisks in the corresponding colour of the relative probe mark the designated N2pc time window of those waveforms where follow-up pairwise Bayesian analysis revealed evidence for contralateral negativity (* = *BF*_10_ > 3 or “substantial evidence”, ** = *BF*_10_ > 10 or “strong evidence”, *** = *BF*_10_ > 30 or “very strong evidence”, **** = *BF*_10_ > 100 or “decisive evidence”). The results of the permutation analyses are also illustrated, with lines in the colour of the corresponding probe indicating the approximate latency and size of the clusters where a reliable contralateral negativity was detected.

As can be seen in Figure 3a, target colour probes increasingly triggered N2pc components towards the end of the preparation period, with the most apparent N2pc at probe 7, as well as an N2pc for probe 1 that immediately followed the task-relevant display. In contrast, non-target colour probes (Figure 3b) did not appear to elicit clear N2pc components. This was reflected by the results of the overall ANOVA. A main effect of Laterality (*F* (1,17) = 21.43, *p* < 0.001, η2G = 0.010) confirmed the presence of reliable probe N2pcs, which was accompanied by a Laterality * Probe Colour interaction (*F* (1,17) = 25.13, *p* < 0.001, η2G = 0.004), reflecting larger probe N2pcs for target colour as compared to non-target colour probes. There was also a Laterality * Probe Time interaction (*F* (6,102) = 3.66, *p* = 0.002, η2G = 0.003), confirming that amplitudes within the N2pc time window differed across the seven consecutive probe displays.

An ANOVA assessing the target colour probes indicated a highly significant main effect of Laterality (*F* (1,17) = 35.88, *p* < 0.001, η2G = 0.025), confirming the presence of reliable probe N2pcs. This was accompanied by a Laterality * Probe Time interaction (*F* (6,102) = 3.44, *p* = 0.004, η2G = 0.006). As can be seen in Figure 3a, N2pc components were maximal for the probe display immediately preceding the task-relevant display (probe 7). The results of the permutation analysis suggested an N2pc was also present for probes 1, 3, 5, and 6. This was confirmed by follow-up pairwise Bayesian comparisons of the contra- and ipsilateral ERPs within the N2pc time window of each probe, demonstrating decisive evidence for the presence the N2pc at probes 1, 6, and 7 (all *BF*_10_ > 103.06, *d* ≥ 0.34), as well as very strong evidence at probe 3 (*BF*_10_ = 48.52, *d* = 0.25) and substantial evidence at probe 5 (*BF*_10_ = 4.02, *d* = 0.29). There was little to no evidence of any lateralisation at probe 2 (*BF*_10_ = 1.88) or probe 4 (*BF*_10_ = 1.41).

For non-target colour probes, the main effect of Laterality did not reach significance (*F* (1,17) = 3.48, *p* = 0.079, η2G = 0.002). While there was a reliable Laterality * Probe Time interaction (*F* (6,102) = 2.79, *p* = 0.02, η2G = 0.004), the permutation analysis only identified a reliable cluster at probe 4, and this was also the only probe where follow-up pairwise Bayesian comparisons of contra- and ipsilateral ERPs within the N2pc time window found substantial evidence for the presence of a N2pc (*BF*_10_ = 6.47, *d* = 0.34). For all other probes, *BF*_10_ values were between 0.5 and 2.3 (*d* ≤ 0.23).^1^

#### 3.3.2 Go/NoGo Task

**Figure 4:**
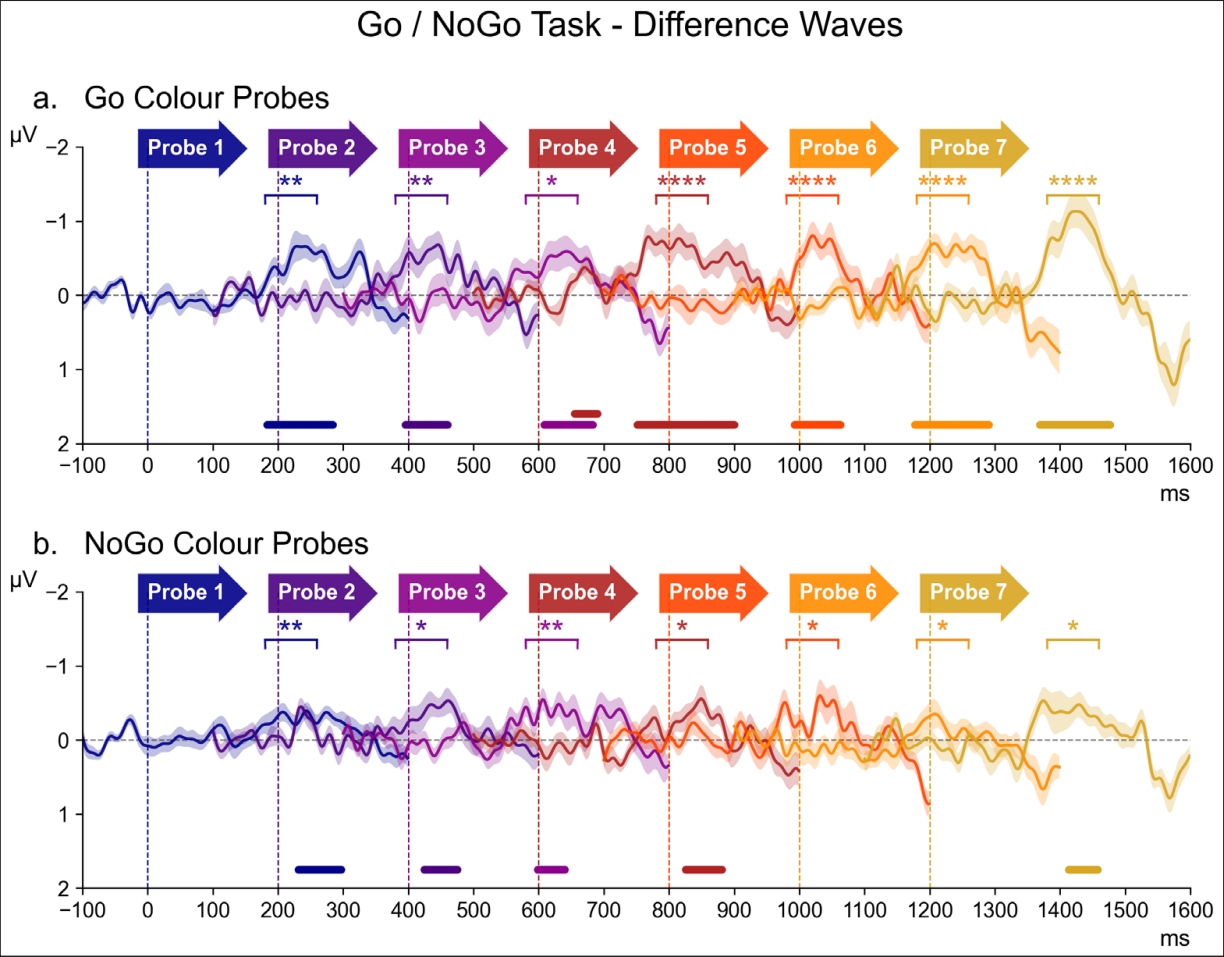
The series of difference waves (contralateral – ipsilateral waveforms) elicited in the Go/NoGo Task over scalp sites PO7/PO8, by target (Go) colour probes (panel a) and non-target (NoGo) colour probes (panel b). The structure and layout of Figure 4 is otherwise identical to that of Figure 3.

Figure 4 shows contralateral/ipsilateral difference waveforms for all successive probe displays in the Go/NoGo task, separately for probes that matched the target (Go) colour or the non-target (NoGo) colour (top and bottom panels; for the source waveforms of these difference waves, see Figure 7 in the appendix). These difference waves are arranged in the same way as in Figure 3, and again also includes the results of both the follow-up pairwise Bayesian comparisons and permutation analyses. Critically, as shown in Figure 4a, Go colour probes triggered clear N2pc components throughout the preparation period, with the most prominent N2pc again for probe 7. For the non-target colour probes (Figure 4b), N2pcs were much smaller but also seemed reliably present.

In the overall ANOVA, a main effect of Laterality (*F* (1,17) = 23.46, *p* < 0.001, η2G = 0.037) demonstrated the presence of reliable probe N2pcs in this non-search task. There was also a Laterality * Probe Colour interaction (*F* (1,17) = 14.28, *p* = 0.001, η2G = 0.004), confirming that Go colour triggered larger N2pcs than NoGo colour probes. In contrast to the Colour Search task, there was no reliable Laterality * Probe Time interaction (*F* (6,102) = 1.61, *p* = 0.2, η2G = 0.001).

An ANOVA of N2pc results from Go colour probes obtained a highly significant main effect of Laterality (*F* (1,17) = 28.93, *p* = 0.001, η2G = 0.062), thus confirming the presence of N2pcs during the preparation period. Even though N2pc components were again seemingly maximal for the probe that immediately preceded the task-relevant display (probe 7), the Laterality * Probe Time interaction did not reach significance (*F* (6,102) = 2.15, *p* = 0.054, η2G = 0.004). The permutation analyses indicated that N2pcs were also reliably present for all earlier probes (see Figure 4, top panel). This was confirmed by follow-up Bayesian comparisons of the contra- and ipsilateral ERPs, which found decisive evidence for the presence of an N2pc for probes 4, 5, 6, and 7 (all *BF*_10_ ≥ 165.75, *d* ≥ 0.51), strong evidence for probes 1 and 2 (all *BF*_10_ ≥ 14.90, *d* ≥ 0.31), and substantial evidence for probe 3 (*BF*_10_ = 6.69, *d* = 0.38).

For NoGo colour probes, there was a reliable main effect of Laterality (*F* (1,17) = 12.24, *p* = 0.003, η2G = 0.018), demonstrating that these probes also triggered N2pc components. There was no Laterality * Probe Time interaction (*F* (6,102) = 0.50, *p* = 0.8, η2G = 0.001). Permutation analyses identified significant clusters of contralateral-ipsilateral differences within the N2pc time-window at probes 1, 2, 3, 4, and 7 (Figure 4b). Follow-up Bayesian comparisons of the contra- and ipsilateral ERPs within the N2pc time window provided strong evidence for the presence of an N2pc for probes 1 and 3 (all *BF*_10_ ≥ 10.85, *d* ≥ 0.20), along with substantial evidence at probes 2, 4, 5, 6, and 7 (all *BF*s ≥ 3.24, *d* ≥ 0.17).

#### 3.3.3 Shape Discrimination Task

**Figure 5:**
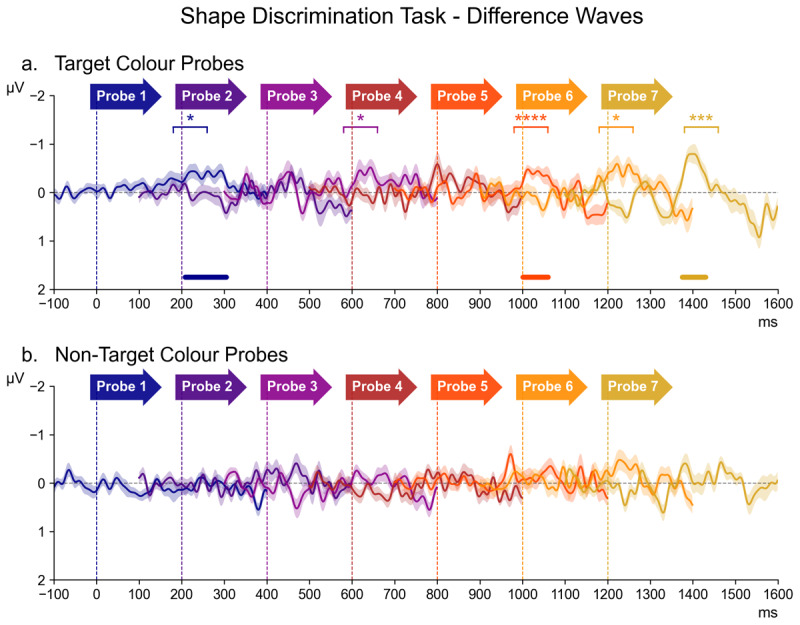
The series of difference waves (contralateral – ipsilateral waveforms) elicited in the Shape Discrimination Task over scalp sites PO7/PO8, by target colour probes (panel **a)** and non-target colour probes (panel b). The structure and layout of Figure 5 is otherwise identical to that of Figure 3.

**Figure 6:**
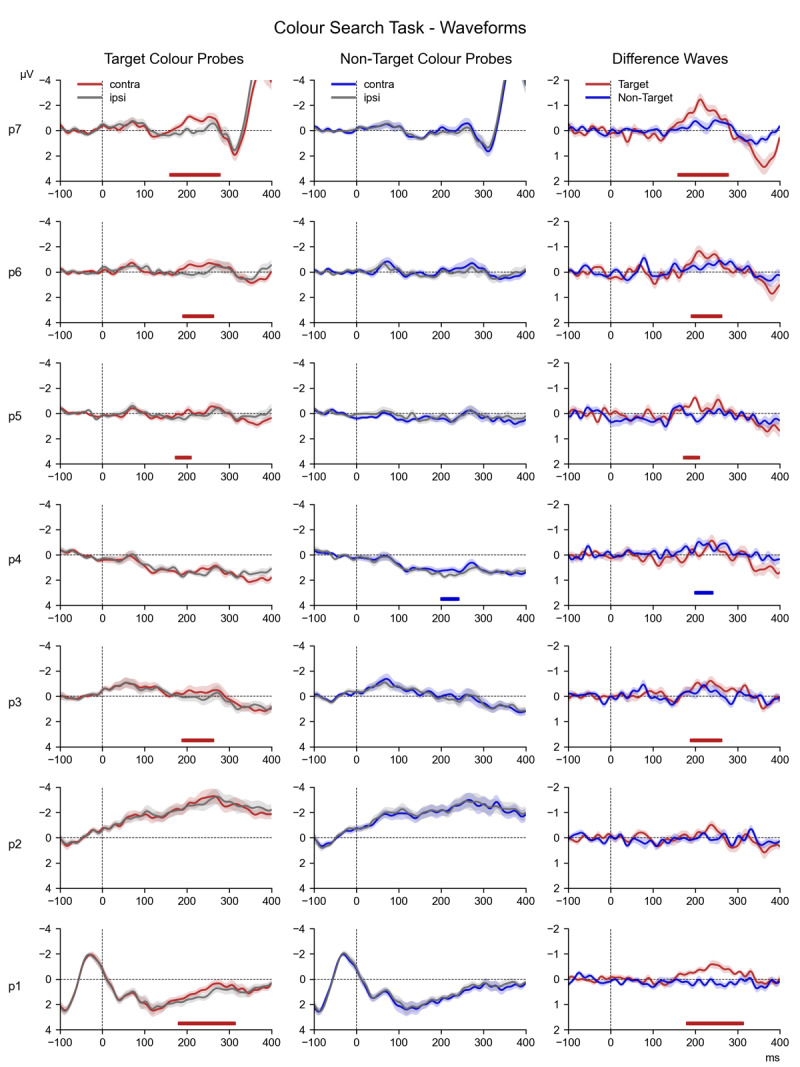
Target Colour Probes (left column) and Non-Target Colour probes (middle column): ERP waveforms elicited over scalp positions PO7/PO8, contralateral (target colour: red, non-target colour: blue) and ipsilateral (grey) to the location of each colour singleton probe. Significant clusters of contralateral negativity detected through permutation analysis appear as a horizontal line at the bottom of each figure (target colour: red, non-target colour: blue). Difference waves: The contralateral – ipsilateral difference waves calculated from the target colour (red) and non-target colour (blue) waveforms for each probe (clusters of contralateral negativity are coloured accordingly).

**Figure 7:**
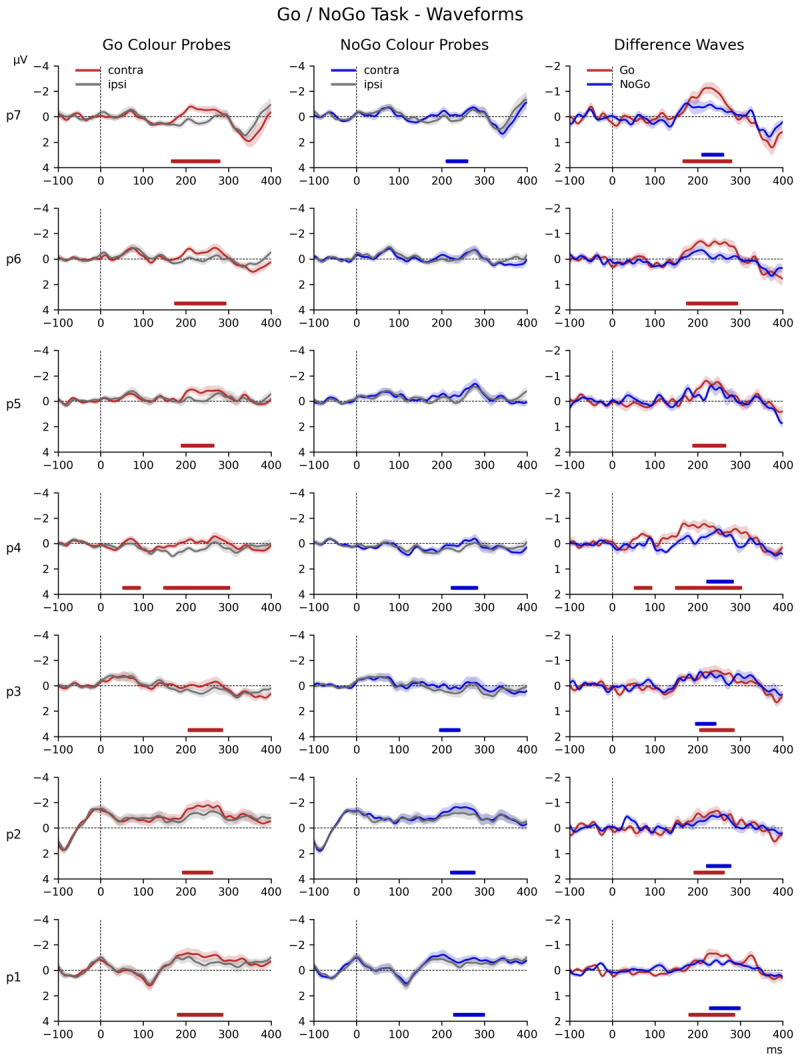
Go Colour Probes (left column) and NoGo Colour probes (middle column): ERP waveforms elicited over scalp positions PO7/PO8, contralateral (Go colour: red, NoGo colour: blue) and ipsilateral (grey) to the location of each colour singleton probe. Significant clusters of contralateral negativity detected through permutation analysis appear as a horizontal line at the bottom of each figure (Go colour: red, NoGo colour: blue). Difference waves: The contralateral – ipsilateral difference waves calculated from the Go colour (red) and NoGo colour (blue) waveforms for each probe (clusters of contralateral negativity are coloured accordingly).

**Figure 8:**
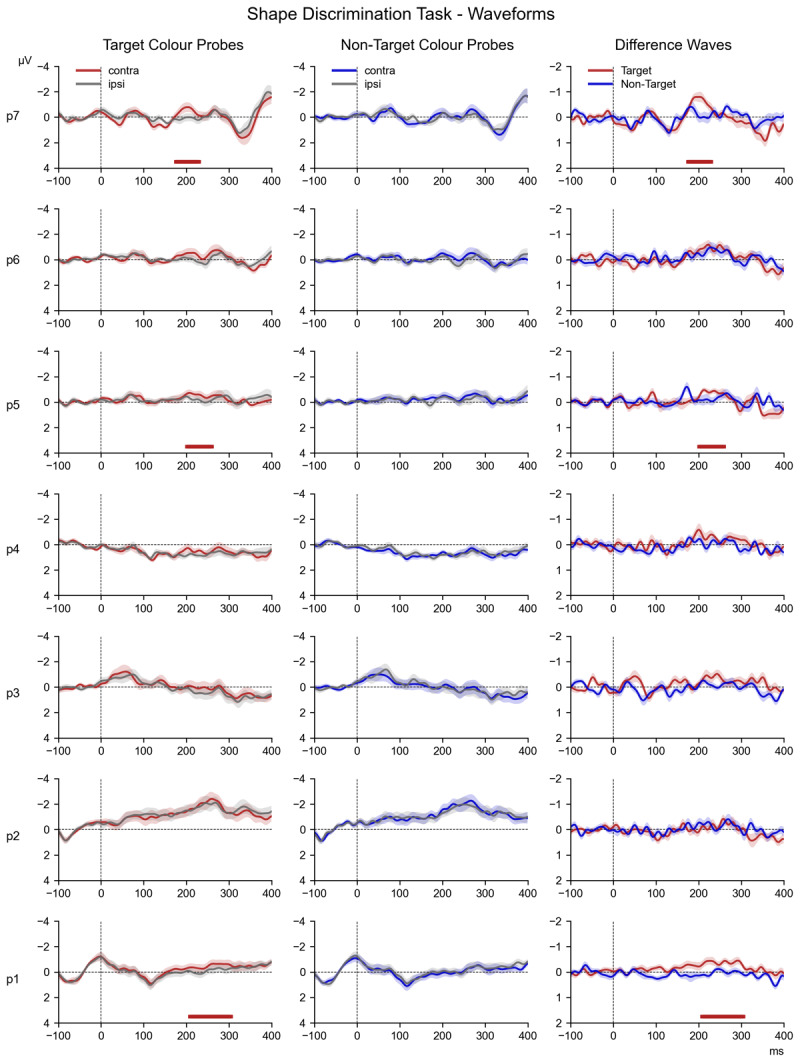
Target Colour Probes (left column) and Non-Target Colour probes (middle column): ERP waveforms elicited over scalp positions PO7/PO8, contralateral (target colour: red, non-target colour: blue) and ipsilateral (grey) to the location of each colour singleton probe. Significant clusters of contralateral negativity detected through permutation analysis appear as a horizontal line at the bottom of each figure (target colour: red, non-target colour: blue). Difference waves: The contralateral – ipsilateral difference waves calculated from the target colour (red) and non-target colour (blue) waveforms for each probe (clusters of contralateral negativity are coloured accordingly).

Figure 5 shows N2pc difference waveforms for probes 1 to 7 in the Discrimination task, separately for the target and non-target colour probes, as well as the follow-up Bayesian comparisons and permutation analyses, arranged in the same way as the probe N2pc results shown in Figures 3 and 4 (for the source waveforms of these difference waves, see Figure 8 in the appendix). These data suggest that target colour probes triggered N2pc components towards the end of the preparation interval, whereas non-target colour probes did not appear to elicit any N2pcs. This was substantiated by the overall ANOVA, which obtained a main effect of Laterality (*F* (1,17) = 8.94, *p* = 0.008 η2G = 0.006) that was accompanied by Laterality * Probe Colour interaction (*F* (1,17) = 13.31, *p* = 0.002, η2G = 0.004). For non-target colour probes, there was no main effect of Laterality (*F* (1,17) = 2.56, *p* = 0.1, η2G = 0.002), nor any Laterality * Probe Time interaction (*F* (6,102) = 0.91, *p* = 0.5, η2G = 0.001), and no sustained clusters of contralateral negativity were identified by the permutation analysis (Figure 5, bottom panel). Further, the follow-up Bayesian comparisons provided no evidence for the presence of N2pcs at any probe (all *BF*_10_ between 0.62 and 1.99, *d* ≤ 0.21).

By contrast, a highly significant main effect of Laterality (*F* (1,17) = 14.09, *p* = 0.002, η2G = 0.012) was present for the target colour probes, without a Laterality * Probe Time interaction (*F* = 1.46, *p* = 0.2, η2G = 0.002). As shown in Figure 5 (top panel), permutation analysis identified three clusters where the N2pc component was likely to be present at probes 1, 5, and 7. Follow-up Bayesian comparisons of contra- and ipsilateral ERPs within the N2pc time window revealed decisive evidence for the presence of an N2pc (*BF*_10_ = 304.41, *d* = 0.28) at probe 5, as well as very strong evidence for an N2pc at probe 7 (*BF*_10_ = 30.74, *d* = 0.35), while also indicating substantial evidence for an N2pc at probes 1, 3, and 6 (all *BF*_10_ ≥ 3.18, *d* ≥ 0.31). No evidence was present at probes 2 (*BF*_10_ = 0.61, *d* = 0.04) or 4 (*BF*_10_ = 2.96, *d* = 0.27).

#### 3.3.4 Between-task analyses

To assess possible differences in the degree to which colour-selective preparation processes were activated between the three tasks, we analysed N2pc components to target colour probe displays observed in the Colour Search, Go/Nogo, and Shape Discrimination tasks, with Task as additional three-level factor. A main effect of Laterality (*F* (1,17) = 32.40, *p* < 0.001, η2G = 0.028) was accompanied by a Laterality * Probe Time interaction (*F* (6,102) = 4.57, *p* < 0.001, η2G = 0.003), reflecting the presence of probe N2pcs that tended to increase towards the end of the preparation period across all three tasks. There was an interaction between Laterality and Task (*F* (2,34) = 6.92, *p* = 0.003, η2G = 0.002), indicating that probe N2pc amplitudes differed between tasks. However, there was no three-way interaction (Laterality × Task × Probe Number: *F* (12,204) = 0.96, *p* = 0.49, η2G < 0.001), which suggests that the time course of template activation did not differ systematically between the three tasks. To further assess between-task N2pc amplitude differences, we focused on N2pc components in response to probes presented immediately prior to the task-relevant display (probe 7) when template activation should be maximal. An overall ANOVA across all three tasks revealed a reliable Laterality * Task interaction (*F* (2, 34) = 6.38, *p* = 0.004, η2G = 0.002), demonstrating that there were indeed task-induced differences in the activation levels of colour templates. To identify the basis of these differences, three separate follow-up ANOVAs were run for each pairwise combination of two tasks (Colour Search * Shape Discrimination, Go/NoGo * Shape Discrimination, and Colour Search * Go/NoGo). Laterality * Task interactions were obtained when the Shape Discrimination task was analysed together with each of the other two tasks (Colour Search * Shape Discrimination: *F* (1,17) = 6.62, *p* = 0.02, η2G = 0.006 | Go/No Go * Shape Discrimination: *F* (1,17) = 10.67, *p* = 0.005, η2G = 0.011), indicating that N2pcs to probe 7 were smaller in the Shape Discrimination task. This was confirmed by follow-up Bayesian comparisons, which obtained substantial evidence for probe N2pc amplitude differences between the Colour Search and Shape Discrimination tasks (*BF*_10_ = 4.04), as well as the Go/NoGo and Shape Discrimination tasks (*BF*_10_ = 8.83). By contrast, there was no Laterality * Task interaction when comparing the Colour Search and Go/NoGo tasks (*F* (1,17) = 0.566, *p* = 0.5, η2G = 0.001).

## 4 Discussion

The goal of the current study was to obtain new insights into the factors that determine whether representations of target-defining features (attentional templates) are activated in a preparatory fashion. It is possible that such proactive mechanisms exclusively operate in situations where templates are needed to guide attention towards targets among multiple distractors in visual search displays. Alternatively, these mechanisms may also be employed in non-search tasks where guidance is not involved, and attentional templates are employed only in the recognition of target objects. To investigate the preparatory activation of templates for guidance and templates for recognition, we measured N2pc components to colour singleton probes that appeared in rapid succession in the interval between two successive task-relevant displays. The presence of N2pcs to a particular probe demonstrates that it had attracted attention, which implies that a corresponding colour-specific template was active at the moment in time when this probe was presented.

The results from the Colour Search task, where colour-defined targets appeared among multiple distractors, confirmed the evidence for the preparatory activation of guidance templates from previous studies (Grubert & Eimer, 2018, 2020). Target colour probes triggered reliable N2pc components which increased in size towards the later parts of the preparation interval, indicating that a target colour guidance template was active during the preparation for search, and that its activation state was modulated in line with temporal expectations about the arrival of the next search display. Importantly, the probe N2pcs in this search task were colour-selective, as demonstrated by an interaction between laterality and probe colour. Non-target colour probes did not elicit overall reliable N2pc components, and further, did not show any increase of N2pcs amplitudes during the preparation period. Only for probe 4, which appeared in the middle of the preparation interval, was there some evidence for the presence of an N2pc.^2^

The critical new question addressed in the present study was whether analogous preparatory processes also operate for target templates in non-search tasks when no attentional guidance is needed. The probe N2pc results obtained in the Go/NoGo task provided an unequivocal answer. In this task, task-relevant objects appeared without competing distractors, and their colour determined whether they required a response. There was a highly significant laterality effect, demonstrating that colour probes did trigger N2pc components during the preparation period. Probes that matched the Go colour elicited reliable N2pcs throughout the interval between two task-relevant displays. These N2pcs again tended to increase in size for probes closer to the next target. Probes that matched the NoGo colour also triggered reliable N2pc components. However, these NoGo colour probe N2pcs showed no increase towards later phases of the task preparation process. Importantly, they were also significantly smaller than the N2pcs for Go colour probes, particularly probes that appeared just prior to the next target. This shows that proactive target template activation processes in this task were colour-sensitive. The template for the target (Go) colour was selectively prioritized, although a NoGo colour template was also activated to some degree. This is not entirely surprising as the NoGo colour was also potentially relevant during response selection, as it indicated that a response had to be withheld. The presence of reliable N2pcs for both Go and NoGo colour probes in this task is interesting because it suggests that two different target templates can be activated concurrently (see Grubert & Eimer, 2020, for corresponding evidence for the simultaneous activation of two guidance templates during search preparation).

In the Shape Discrimination task, neither guidance templates nor colour-specific target recognition templates were needed for successful performance, as target objects again appeared without distractors, always in the same colour, and response selection was determined by their orientation. Therefore, no probe N2pcs indicative of preparatory colour template activation was expected in this task. In fact, probes that matched the constant target colour elicited an overall significant N2pc, indicating that a corresponding colour template was activated during the preparation period. Thus, the fact that the colour of target objects was constant was sufficient for participants to prepare for this colour, even though it was not necessary for target recognition and response selection. However, these target colour probe N2pcs were smaller and less reliably present across all probe positions relative to the N2pcs triggered by target colour and Go colour probes in the Colour Search and Go/NoGo tasks, respectively. Non-target colour singleton probes in the Shape Discrimination task altogether failed to trigger any reliable N2pcs, demonstrating again that probe N2pcs were colour-selective. This is important, because it demonstrates that these N2pcs do not simply reflect the stimulus-driven exogenous capture of attention by a salient colour singleton in the probe display but are instead associated with the endogenous preparatory activation of colour-specific attentional templates.

To identify task-dependent differences of proactive template activation at the point in time when these templates are maximally active, we compared N2pcs to the target colour probe that appeared immediately before the task-relevant display between the three tasks. Probe N2pc amplitudes were reliably smaller in the Shape Discrimination task where colour was not needed for either guidance or recognition relative to the other two tasks, demonstrating that proactive colour template activation is sensitive to task relevance. In contrast, there was no difference between N2pc components to target colour probes in the Colour Search task and N2pcs to Go colour probes in the Go/NoGo task. This suggests that at least in the context of the present task manipulations, colour-selective guidance and target templates were activated to a similar degree during task preparation.

The goal of the current study was to find out whether proactive template activation processes are exclusively triggered in search tasks where attentional guidance is needed to rapidly locate target objects among competing distractors. Our results clearly show that this is not the case, as clear probe N2pc components indicative of colour template activation were present during the preparation for non-search tasks where task-relevant objects appeared without concurrent distractor objects, and guidance was not required. These observations from the Go/NoGo task demonstrate for the first time that, similar to guidance templates, target templates required to discriminate task-relevant objects and subsequent response selection are also triggered in a proactive fashion. The finding that small but reliable target colour probe N2pcs were also present in the Shape Discrimination task was surprising, given that target colour was constant and thus not nominally task-relevant. It is possible that in the target templates for this task, target colour was represented together with the task-relevant orientation (horizontal/vertical), and thus triggered some attentional capture by colour-matching probes. This could suggest that these templates are object-based (i.e., representing the orientation of coloured bars) rather than purely feature-based, and this possibility needs to be investigated further.

This conclusion that both guidance and target templates are activated in a preparatory fashion is important for current proposals about the fundamental difference between these two types of templates (e.g., Wolfe, 2021). Given that they have different functions and operate at different points in time during the processing of visual input (either before or after the selection and encoding of candidate target objects), it would have been plausible to assume that they also differ systematically with respect to their temporal activation profile, with preparatory template activation only triggered in tasks where the rapid guidance of attention is required. The current findings show that the preparatory activation of attentional templates is not exclusively associated with the need for guidance in visual search. They suggests that search templates might be most appropriately conceived as generic representations of relevance, regardless of whether their relevance is related for target-nontarget discrimination or to target identification and response selection processes.

This does however not imply that target template activation is always elicited in a proactive fashion in all task contexts. In the Go/NoGo task of the present study, target recognition was based on a simple colour discrimination, and it is possible that more complex target templates (e.g., when target objects are defined at the category level, or as members of a particular memory set) are primarily triggered reactively. Along similar lines, the preparatory activation of colour templates in all three tasks may be linked to the fact that probe displays were task-irrelevant, so that there were no additional search-unrelated task demands during the preparation period. An interesting question for future research is whether such template activation processes remain active even when such competing task demands are present (e.g., in a dual-task context). It is possible that in such circumstances, only templates that are required for the guidance of search are triggered proactively.

More generally, it should be noted that the current evidence for the preparatory activation of colour-selective templates for target recognition in the Go/NoGo task (and to some degree in the Shape Discrimination task) was obtained when no guidance of attention was required. These tasks were chosen specifically to observe the activation of target templates in isolation, in the absence of any concurrently active guidance templates. However, in many other contexts, such as in typical visual search tasks, both guidance and target templates are involved, and operate sequentially to ensure correct performance. In such tasks, guidance templates are activated prior to the presentation of a search display, but it is an open question whether target templates are proactively triggered as well. The important question whether guidance and target templates can be activated concurrently during task preparation will also need to be addressed in future research.

## Data Accessibility Statement

The per-participant EEG and behavioural data, associated analysis scripts, as well as the mean amplitudes used for the EEG data reported here have all been anonymised and made available in an OSF repositotry at the following DOI: 10.17605/OSF.IO/BXE93.

For the purposes of open access, the author has applied a creative commons attribution (CC BY) licence to any author accepted manuscript version arising.

## Footnotes

To assess whether N2pcs to target colour probes in the Colour Search task were affected by colour priming from the immediately preceding probe display, in line with the idea that feature-based attentional allocation processes are modulated by selection history (Awh et al., 2012), we conducted an additional analysis with Selection History (probe colour repetition versus change) as additional factor. There was no main effect of Selection History on mean probe N2pc amplitude and no interaction between Selection History and Probe time (both F < 1.1), which strongly suggests that there was no colour priming effect between successive probe displays.

This moderate evidence for a small residual N2pcs to a non-target colour probe differ from a previous study (Grubert & Eimer, 2018), where these probes did not elicit any N2pcs throughout the preparation period. It is possible that this difference was due to the inclusion of two non-search tasks in the current study, which may have slightly reduced the colour selectivity of guidance templates in the Colour Search task.
